# Sound Waves Promote *Arabidopsis thaliana* Root Growth by Regulating Root Phytohormone Content

**DOI:** 10.3390/ijms22115739

**Published:** 2021-05-27

**Authors:** Joo Yeol Kim, Hyo-Jun Lee, Jin A Kim, Mi-Jeong Jeong

**Affiliations:** 1 Department of Agricultural Biotechnology, National Institute of Agricultural Science, Rural Development Administration, 370 Nongsaengmyoeng-ro, Deokjin-gu, Jeonju, Jeollabuk-do 54874, Korea; rlawnduf@korea.kr (J.Y.K.); jakim72@korea.kr (J.A.K.); 2 Plant Systems Engineering Research Center, Korea Research Institute of Bioscience and Biotechnology (KRIBB), 125 Gwahak-ro, Yuseong-gu, Daejeon 34141, Korea; hyojunlee@kribb.re.kr; 3 Department of Functional Genomics, KRIBB School of Bioscience, University of Science and Technology, Daejeon 34113, Korea

**Keywords:** sound wave, root growth promotion, *Arabidopsis thaliana*, auxin, cytokinin

## Abstract

Sound waves affect plants at the biochemical, physical, and genetic levels. However, the mechanisms by which plants respond to sound waves are largely unknown. Therefore, the aim of this study was to examine the effect of sound waves on *Arabidopsis thaliana* growth. The results of the study showed that *Arabidopsis* seeds exposed to sound waves (100 and 100 + 9k Hz) for 15 h per day for 3 day had significantly longer root growth than that in the control group. The root length and cell number in the root apical meristem were significantly affected by sound waves. Furthermore, genes involved in cell division were upregulated in seedlings exposed to sound waves. Root development was affected by the concentration and activity of some phytohormones, including cytokinin and auxin. Analysis of the expression levels of genes regulating cytokinin and auxin biosynthesis and signaling showed that cytokinin and ethylene signaling genes were downregulated, while auxin signaling and biosynthesis genes were upregulated in *Arabidopsis* exposed to sound waves. Additionally, the cytokinin and auxin concentrations of the roots of *Arabidopsis* plants increased and decreased, respectively, after exposure to sound waves. Our findings suggest that sound waves are potential agricultural tools for improving crop growth performance.

## 1. Introduction

Plants respond continually to biotic and abiotic stimuli, as well as to various external signals such as light, wind, and sound [1,2]. Plants modulate organ formation and growth to adapt to changing environmental conditions. Roots support plants in the soil and absorb the necessary nutrients and water for plant growth and development. Promoting root growth improves plant anchorage and enhances nutrient and water uptake, which increases biotic and abiotic stress resistance and improves crop productivity and quality [3,4]. Therefore, several studies have aimed at improving crop growth performance by improving root development and growth [5,6].

Plant root growth and development are regulated by a balanced interaction of various phytohormones, including auxin and cytokinin, which regulate cell division, differentiation, and elongation in the root meristem [7,8]. Although auxin acts synergistically with cytokinin in the shoots, the hormones act antagonistically in the roots to maintain the size of the root meristem and the specification of the root stem cell niche [9,10]. An auxin concentration gradient is maintained in the root meristem via auxin biosynthesis and transport. Auxin transport is primarily regulated by auxin resistant 1 (AUX1) (an influx carrier) and PINs (efflux carriers) [11,12]. AUX1 regulates auxin uptake in *Arabidopsis* roots. Auxin transported from shoots to roots by PINs forms a reflux loop in the roots [13]. Auxin concentration is highest near the quiescent center (QC) and root cap, where it induces cell division and increases meristem size [14]. In *Arabidopsis thaliana*, cytokinin is recognized via three cytokinin receptors (AHK2, AHK3, and AHK4), which carry signals to the nucleus and activate type-B response regulators (ARRs) [15,16]. ARR1, ARR10, and ARR12 affect cell division in the root meristem. These regulators are specifically expressed in the root transition zone and act during early and late meristem development [9]. In particular, the cytokinin signaling pathway proteins, AHK3, ARR1, and ARR12 activate SHY2 in the root, which negatively controls *PIN* expression to regulate auxin transport. These events modulate the ratio between auxin and cytokinin to determine root meristem size [17,18].

Ethylene also interacts with other phytohormones during primary root growth in *Arabidopsis* [19,20]. Ethylene regulates root growth by controlling auxin biosynthesis, transport, and signaling in the root meristem. In this process, EIN3 and ERF1 link ethylene signaling with auxin signaling [21,22]. Regulation of root growth by cytokinin is also partially affected by the ethylene-related signaling components EIN1 and EIN2 [23]. Additionally, ethylene regulates cell division in roots by controlling the expression of genes encoding cyclins (CYCs) according to developmental stage, hormonal status, and environmental conditions [23,24].

Sound is defined as acoustic energy in the form of molecular vibrations transmitted through various matter forms, such as gases, liquids, and solids. Sound waves are characterized by frequency (Hz) and intensity (dB). Several studies have examined the effects of sound waves on animals, but recently, it has been revealed that plants also respond to sound waves. Our previous studies showed that sound waves can improve crop quality by regulating genes involved in the metabolism of functional substances. For example, in rice (*Oryza sativa*), experiments using a β-glucuronidase (GUS) reporter system demonstrated that sound waves affect the promoter activity of the alcohol dehydrogenase (*Ald*) gene [25]. In tomato (*Solanum lycopersicum*), treatment with 1 kHz sound waves delays fruit ripening by regulating the expression of genes related to ethylene biosynthesis [26]. We also reported that sound waves increase the antioxidant content of sprout vegetables by regulating the expression of genes related to vitamin C and flavonoid biosynthesis [27,28]. Other studies have reported that sound waves influence plant growth and hormone levels. Sound wave treatment significantly increases the growth rate of *Chlorella* by 12–30% [29], and promotes growth and germination of rice and cucumber (*Cucumis sativus*) seeds [30,31]. The hypocotyl elongation of *Arabidopsis* seedlings is also enhanced by sound wave treatment [32]. Additionally, sound waves increase indole acetic acid (IAA) levels, decrease abscisic acid (ABA) levels in chrysanthemum, and induce IAA, gibberellin (GA), jasmonic acid (JA), and salicylic acid (SA) production in *Arabidopsis* [33,34]. However, the effects of sound waves on the growth of other tissues, including roots, remain largely unknown.

Therefore, the aim of this study was to examine the effect of sound waves on *Arabidopsis* root growth and development. Furthermore, we identified the phytohormones and genes regulating *Arabidopsis* root growth and development.

## 2. Results

### 2.1. Seed Exposure to Sound Waves Promotes Arabidopsis Root Growth

**Figure 1:**
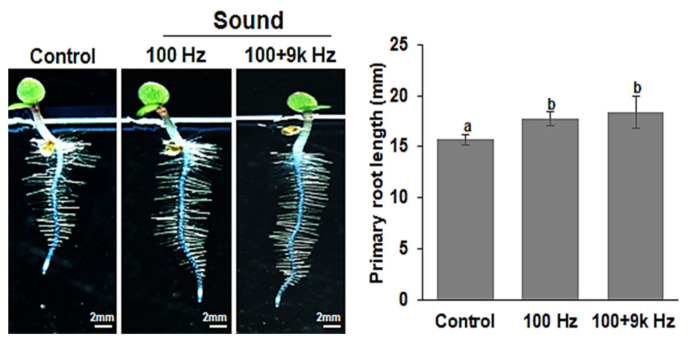
Sound wave treatment promotion of *Arabidopsis thaliana* root growth. Seeds were exposed to 100 or 100 + 9k Hz sound waves and seedlings were observed on the 5th day after sowing using an optical microscope (×10; **left panel**). The graph shows the average primary root length for each treatment (± standard error). Different letters above the bars indicate significantly different mean values (*p* < 0.05) based on Duncan’s multiple range test. Scale bars represent 2 mm. Images show representative individuals of at least 50 seedlings in three or more replicate experiments.

Our preliminary work showed that exposure of pumpkin seeds to 9 kHz sound waves for 5 h daily for 5 day promoted root growth (Figure S1). However, the optimum sound frequency for growth promotion depends on the plant species, exposure time, and growth stage. In the present study, *Arabidopsis* seeds were exposed to sound waves under various conditions (frequency, exposure time, and growth stage) (Figure S2). The results of the study showed that *Arabidopsis* seeds exposed to sound waves (100 and 100 + 9k Hz) for 15 h per day for 3 day had significantly longer root growth than that in the control group. However, the *Arabidopsis* exposed to the dual-frequency treatment (100 + 9k Hz) had slightly longer root growth than that in the single frequency treatment (100 Hz). Analysis of root length showed that the *Arabidopsis* in the 100 (17.68 ± 0.72 mm) and 100 + 9k Hz (18.42 ± 1.25 mm) treatment groups had significantly longer root lengths than those in the control group (15.70 ± 0.50 mm) (Figure 1). This result suggests that 100 and 100 + 9k Hz sound waves have positive effects on early root growth in *Arabidopsis*.

### 2.2. Seed Exposure to Sound Waves Increases Cell Length and Number in Arabidopsis Root Meristem

**Figure 2:**
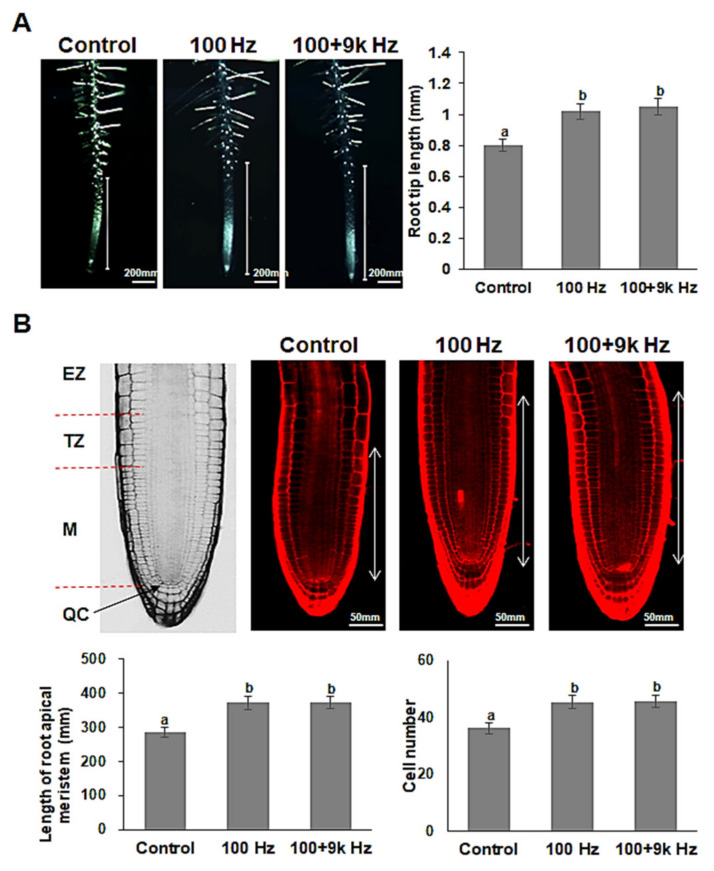
Effect of sound wave treatments on root tip growth and length and cell number of root apical meristem in *Arabidopsis thaliana*. (**A**) Measurement of root tip length by optical microscopy (×60) at the 5th day after sowing of wild type (Col-0) seed treated with or without sound waves. (**B**) Observation of the root apical meristem (M + TZ) and measurement of the length and cell number in this region by confocal microscopy. QC; Quiescent center, M; Meristem, TZ; Transition zone, EZ; Elongation zone. Values represent the means of three measurements ( ± standard error). Different letters above the bars indicate significantly different mean values (*p* < 0.05) based on Duncan’s multiple range test. Scale bars represent (**A**) 200 µm and (**B**) 50 µm. This experiment was conducted at least three times, and each experiment measured 15–20 seedlings.

The effect of sound waves on the growth and cell count of different parts of the roots was also examined using an optical microscope. The root tip was measured from where the root hairs started to develop to the end of the root. The results showed that the *Arabidopsis* in the 100 (1.02 ± 0.15 mm) and 100 + 9k Hz (1.05 ± 0.17 mm) treatment groups had significantly longer root tip lengths than those in the control group (0.80 ± 0.12 mm) (Figure 2A). Additionally, the effect of sound waves on root tip growth was examined at the cellular level using propidium iodide (PI) stain and a confocal microscope. There was a significant increase in the root apical meristem length (from the QC to the first elongated cortex cell) and cell number in this region in the *Arabidopsis* exposed to sound waves. The mean root apical meristem length of the *Arabidopsis* in the control group was 284.6 ± 21.3 µm, while that of the *Arabidopsis* in the 100 and 100 + 9k Hz treatment groups was 371.6 ± 26.4 and 373.4 ± 25.7 µm, respectively. The mean root apical meristem number of the *Arabidopsis* in the control group was 36 ± 3.4, while that of the *Arabidopsis* in the 100 and 100 + 9k Hz treatments was 45 ± 3.2 and 46 ± 4.1 cells, respectively. The increase in root tip growth could explain the overall increase in root length of the *Arabidopsis* exposed to sound waves, indicating that sound waves positively affect cell division to facilitate root growth.

### 2.3. Expression Profiles of Cell Division Related Genes in Arabidopsis Roots are Regulated by Sound Waves

**Figure 3:**
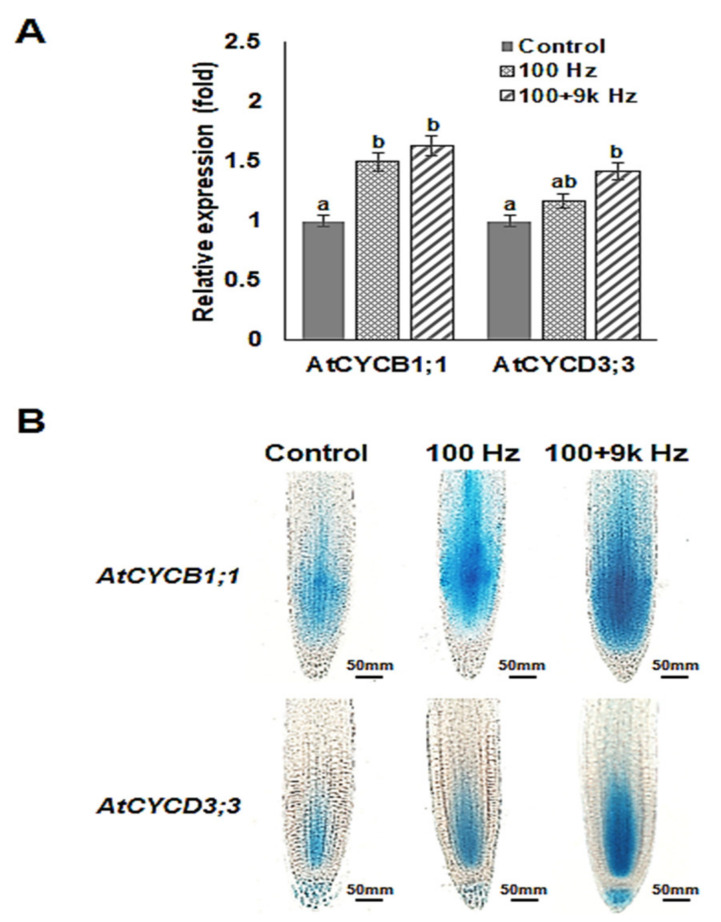
Effect of sound wave treatments on the expression profiles of cell division genes in *Arabidopsis* roots. (**A**) Expression of cyclin genes after sound wave treatments, as determined by qRT-PCR. Values were normalized to the value of the control. Error bars indicate the standard error of the mean of three biological replicates. Different letters above the bars indicate significantly different mean values (*p* < 0.05) based on Duncan’s multiple range test. (**B**) Promoter activity was assessed by GUS staining of *Arabidopsis* plants transformed with GUS reporter gene fusions to the indicated gene promoters. Scale bars represent 50 µm. This experiment was conducted in triplicate, and each experiment measured 25–30 seedlings.

Furthermore, the expression profiles of genes involved in cell division (*AtCYCB1;1* and *AtCYCD3;3*) in the roots of *Arabidopsis* exposed to sound waves were examined (Figure 3). There was a significant increase in the expression of *AtCYCB1;1* and *AtCYCD3;3* in the roots of the *Arabidopsis* in the 100 and 100 + 9k Hz treatments compared with that of the *Arabidopsis* in the control group (Figure 3A). Particularly, the expression of *AtCYCB1;1* increased more than 1.5-fold in the roots of the *Arabidopsis* exposed to sound waves compared with that in the *Arabidopsis* in the control group, indicating that sound wave treatment of seeds regulates the expression of genes involved in cell division in roots after germination.

To confirm the results of the quantitative real-time PCR (qRT-PCR) and to further investigate the effect of sound waves on the expression profiles of cell division genes, we examined the expression and activities of AtCYCB1;1:GUS and AtCYCD3;3:GUS promoters in the roots of the experimental plants using a histochemical assay. Transgenic seeds containing the promoters were exposed to 100 and 100 + 9k Hz sound waves. After treatment, the seeds were grown for 5 d before GUS staining of the roots. There was a significant increase in the expressions and activities of AtCYCB1;1:GUS and AtCYCD3;3:GUS promoters in the root apical meristem of the *Arabidopsis* exposed to sound waves compared to those in the root apical meristem of the *Arabidopsis* in the control group (Figure 3B), which was consistent with the increased cell numbers in the root apical meristem of the sound wave-treated roots (Figure 2B). This result suggests that sound waves activate cell division in the root apical meristem to enhance root growth.

### 2.4. Expression Profiles of Phytohormone-Related Genes in Arabidopsis Roots are Regulated by Sound Waves

**Figure 4:**
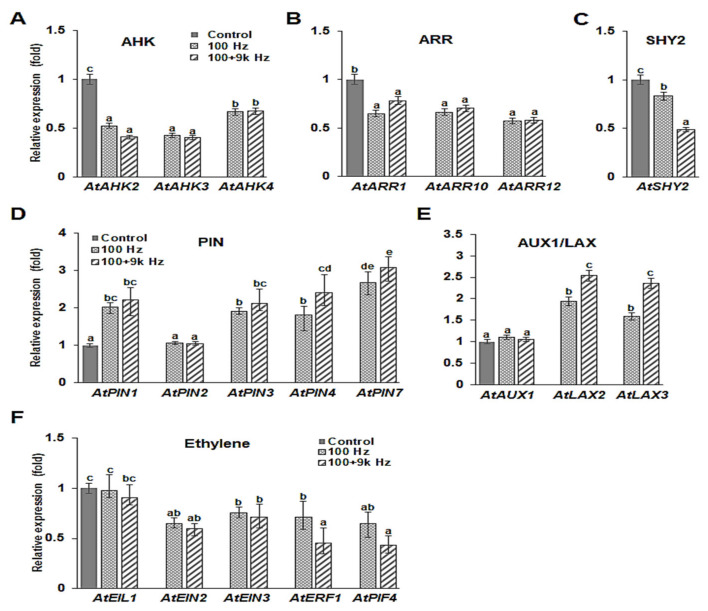
Effect of sound wave treatments on the expression of hormone-related genes in *Arabidopsis* roots. qRT-PCR was used to determine the expression levels of genes related to cytokinin ((**A**), *AHKs*; (**B**), *ARRs*), auxin ((**D**), *PINs*; (**E**), *AtAUX1*, *AtLAX2*, and *AtLAX3*), and ethylene ((**F**), *AtEIL1*, *AtEIN2*, *AtEIN3*, *AtERF1*, and *AtPIF4*) pathways, as well as *AtSHY2* (**C**). The values were normalized to the value of the control. qRT-PCR error bars indicate the standard error of the mean of three biological replicates. Different letters above the bars indicate significantly different mean values (*p* < 0.05) based on Duncan’s multiple range test.

Plant growth is closely related to phytohormone levels and activities, which are affected by environmental stimuli [35]. Phytohormones regulate root growth by modulating cell division [8]. Cytokinin inhibits root growth (Růžička et al., 2009; Moubayidin et al., 2010), and its effects are mediated by cytokinin receptors and response regulators (ARRs) [15,16]. Therefore, we examined the expression profiles of genes regulating some phytohormones, including auxins, cytokinin, and ethylene, in the root meristem of *Arabidopsis* exposed to sound waves (Figure 3). The results showed that there was a significant decrease in the expression of *AtAHK2*, *AtAHK3*, and *AtAHK4* in the roots following exposure to sound waves (Figure 4A). Similarly, sound wave exposure slightly reduced the expression of *AtARR1*, *AtARR10*, and *AtARR12* in the roots (Figure 4B). SHY2 is an auxin signaling repressor belonging to the Aux/IAA gene family, and its expression is regulated by cytokinin signaling [17,18]. There was a significant decrease in *AtSHY2* expression in the roots exposed to the 100 and 100 + 9k Hz sound wave treatments; however, the effect of the 100 + 9k Hz sound wave was more severe, suggesting that the 100 + 9k Hz sound wave was more effective at suppressing *AtSHY2* gene expression (Figure 4C). Next, we examined the expression profiles of genes involved in auxin transport, which is related to root growth [12,36]. The expression of *AtLAX2*, *AtLAX3*, *AtPIN1*, *AtPIN3*, *AtPIN4*, and *AtPIN7* was significantly enhanced by sound wave treatment, whereas that of *AtPIN2* and *AtAUX1* was not significantly affected (Figure 4D,E). Particularly, the expression of *AtLAX2* and *AtPIN7* increased by approximately 3-fold in the sound wave-treated roots compared with that in the untreated roots. Moreover, the 100 + 9k Hz treatment was more effective than the 100 Hz treatment in inducing *PIN* gene expression. To investigate the role of auxin transport in sound wave-induced root growth, *Arabidopsis* wild-type seeds were subjected to the same sound wave treatments and root growth was observed after treatment with 20 and 50 µM of N-1-naphthylphthalamic acid (NPA), an auxin transport inhibitor [37]. We found that pre-germination sound wave treatment did not affect root length in the NPA-treated seedlings (Figure S3). To verify this result, we used *pin3 pin4 pin7* triple mutants that exhibit highly reduced auxin transport activity [13]. Consistent with the inhibitor assays, the pre-germination sound wave-treated seedlings showed similar root lengths to those of the untreated seedlings (Figure S4). This result indicates that pre-germination sound wave treatment activates auxin transport by increasing the expression of auxin transport genes to promote root growth.

Recently, it has been reported that transcription factors of the ethylene signaling pathway, such as *EIN2*, *EIN3*, *EIL1*, *ERF1*, and *PIF4*, act as crosstalk nodes between ethylene and auxin in root growth [22,38,39]. Therefore, we analyzed the expression of these genes in the sound-wave-treated roots by qRT-PCR. The expression of the ethylene-related genes, except *AtEIL1*, was reduced by sound wave treatment. Notably, *AtERF1* and *AtPIF4* expression was further suppressed by treatment with 100 + 9k Hz (Figure 4F). These results suggest that ethylene signaling genes might be related to enhanced root growth by pre-germination sound waves, possibly through interactions with other hormones.

### 2.5. Cytokinin and Auxin Biosynthesis in Arabidopsis Roots are Regulated by Sound Waves

**Figure 5:**
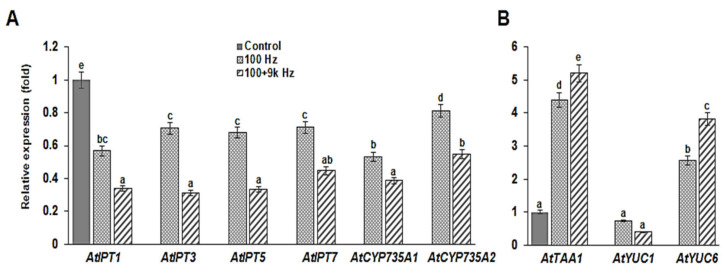
Effect of sound wave treatments on the expression of genes related to cytokinin (CK) and auxin biosynthesis in *Arabidopsis* roots. (**A**) Expression of CK biosynthesis-related genes after sound wave treatments, as determined by qRT-PCR. (**B**) Expression of auxin biosynthesis-related genes after sound wave treatments, as determined by qRT-PCR. Values were normalized to the value of the control. Error bars indicate the standard error of the mean of three biological replicates. Different letters above the bars indicate significantly different mean values (*p* < 0.05) based on Duncan’s multiple range test.

To further understand the effect of sound waves on cytokinin and auxin concentrations and activities in the roots of *Arabidopsis*, the expression profiles of genes regulating cytokinin and auxin biosynthesis were examined. The expression of isopentenyl transferase (*AtIPTs*) and *cytochrome P450 monooxygenase* (*AtCYP735As*), which are representative genes involved in cytokinin biosynthesis in *Arabidopsis* roots [40], was significantly reduced by the sound wave treatments (Figure 5A). Notably, the 100 + 9k Hz sound wave was more effective than the 100 Hz sound wave in suppressing cytokinin biosynthesis gene expression (Figure 5A). Next, we analyzed the auxin biosynthesis genes, tryptophan aminotransferase of *Arabidopsis 1* (*AtTAA1*), and *YUCCAs* (*AtYUC1* and *AtYUC6*) [41]. Although the expression of *AtTAA1* and *AtYUC6* was significantly increased by sound wave treatment, that of *AtYUC1* was unaffected (Figure 5B). These results indicate that the expression of biosynthetic and signaling pathway genes for cytokinin and auxin is regulated by specific sound wave treatments. To investigate the role of auxin biosynthesis in sound wave-induced root growth, pre-germination sound wave-treated *Arabidopsis* seedlings were grown on media containing 20 and 50 µM yucasin (5-(4-chlorophenyl)-4H-1,2,4-triazole-3-thiol), an auxin biosynthesis inhibitor [42]. The effects of sound waves on the seedlings under yucasin treatment were not significantly different (Figure S5), suggesting that *YUCCA*-mediated auxin biosynthesis is involved in sound wave-induced root growth.

**Figure 6:**
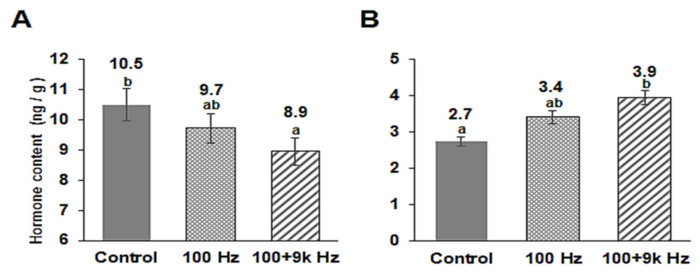
Analysis of plant growth hormone content in *Arabidopsis* roots after sound wave treatments. (**A**) Cytokinin zeatin content. (**B**) Auxin IAA content. Error bars indicate the standard error of three biological replicates. Different letters above the bars indicate significantly different mean values (*p* < 0.05) based on Duncan’s multiple range test.

To examine whether the altered expression of cytokinin and auxin biosynthetic genes affects the phytohormone content in the roots, we determined the zeatin and IAA contents of the roots using liquid chromatography-mass spectrometry (LC-MS). While the 100 Hz sound wave slightly affected the phytohormone contents of the roots, the 100 + 9k Hz sound wave significantly reduced the zeatin and increased the IAA contents of the roots (Figure 6). The root zeatin content of the *Arabidopsis* in the control group was 10.5 ng/g, while that of the *Arabidopsis* in the 100 and 100 + 9k Hz treatments was 9.7 and 8.9 ng/g, respectively (Figure 6A). The root IAA content of the *Arabidopsis* in the control group was 2.7 ng/g, while that of the *Arabidopsis* in the 100 and 100 + 9k Hz treatments was 3.4 and 3.9 ng/g, respectively (Figure 6B).

**Figure 7:**
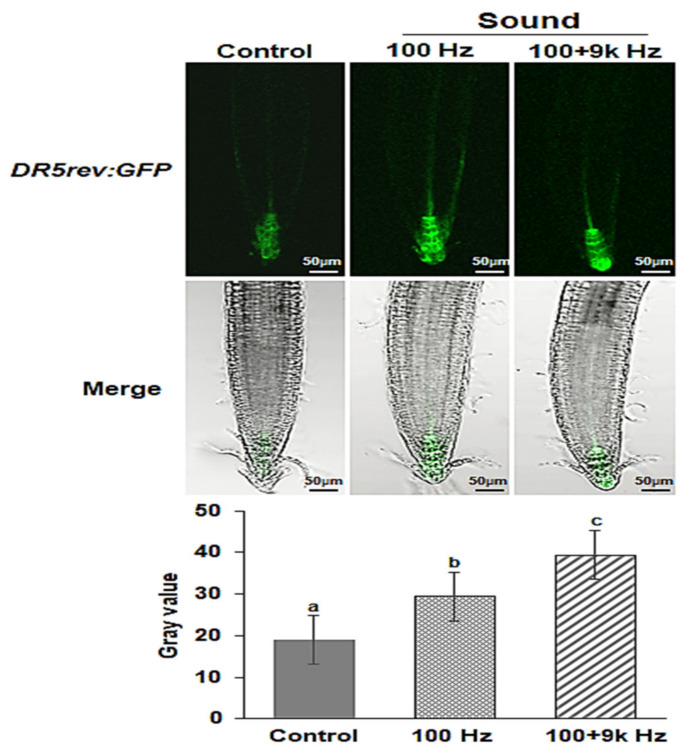
The expression profile of the auxin-specific reporter gene DR5 as green fluorescence in *Arabidopsis* roots exposed to sound wave treatments using confocal microscopy. Images show representative individuals of at least 20 seedlings in three replicate experiments. Scale bars represent 50 µm. Different letters above the bars indicate significantly different mean values (*p* < 0.05) based on Duncan’s multiple range test.

To verify the increased auxin content in the roots due to pre-germination sound waves, we observed the expression of GFP signals in the transgenic seedlings. Exposure of seed to sound waves significantly increased the GFP signals near QC cells (Figure 7).

We next used cytokinin and auxin receptor mutants to examine the role of cytokinin and auxin signaling in sound-wave-induced root growth. Root growth was enhanced in the *Arabidopsis* cytokinin receptor mutants ahk3-1 and ahk4 (Figure S6), whereas the auxin receptor single mutant tir1-1 reduced the difference in root growth compared to that in the untreated control, and the tir1-1 afb2-3 double mutant exhibited similar root growth after sound wave treatment to that of the *Arabidopsis* in the control group (Figure S7).

## 3. Discussion

Roots provide anchorage for terrestrial plants, absorb water and minerals and conduct them to the shoot, and act as nutrient storage organs. Therefore, promoting root growth enhances the overall growth and development of plants in several ways, such as providing better support for plants, increasing nutrient absorption, and reducing stress-related damages [8]. Recently, it has been reported that light perceived by *Arabidopsis* leaves is transmitted via the stem to the root through the vascular bundle. This process also affects root growth and development [43]. Root growth is controlled by several environmental factors as well as the soil. Plant growth and development are constantly adjusted to variable environment conditions through complex interactions between intra-plant signaling and various external stimuli.

Sound waves are a type of external signals that can affect plant growth and development [32,44]. Plants can distinguish between the chewing sounds produced by herbivores and the buzzing sounds of pollinating bees and react accordingly [45,46]. Additionally, constant forces such as touch, wind, and gravity influence plant shape through changes in gene expression [47]. Recently, transcriptomic and proteomic analyses have shown that sound waves can exert positive effects on plant growth [34].

As plants can distinguish between different types of sound waves, it is probable that different frequency ranges may have specific effects on growth and development. However, the optimal sound frequency for any given effect depends on the crop type, exposure time, and application duration; moreover, the mechanisms of sound effects on plants are unclear [48]. In the present study, *Arabidopsis* seeds were exposed to sound waves at two frequencies (100 and 100 + 9k Hz) to determine the appropriate frequency to promote root growth and development. The findings of the study showed that 100 and 100 + 9k Hz treatments were quite effective in improving root growth and development (Figure 1). Increased root growth was accompanied by an increase in the size of the root tip and the number of cells and length of the root apical meristem (Figure 2). These results indicate that exposing *Arabidopsis* seeds to specific sound waves promotes root growth by increasing the length and number of cells in the root apical meristem at the seedling stage.

Generally, the elongation of the length and increase in the number of cells in the root apical meristem are caused by cell division [49]. There was a significant increase in the expression of genes related to cell division in the roots of the *Arabidopsis* exposed to 100 and 100 + 9k Hz sound waves (Figure 3A). Similarly, sound waves increase the cell number in the S phase of chrysanthemum development, suggesting that sound waves promote growth by affecting the cell cycle [50]. Cell cycle progression is controlled by cyclin. In roots, growth and development are regulated by the expression of cyclin genes, particularly *CYCB1;1,* which is a marker for cell division in the root meristem [51]. *Arabidopsis* D-type cyclins are involved in cytokinin response, and *CYCD3;3* stimulates cell division in roots during the formation of the columella and prevents columella stem cell (CSC) differentiation to maintain the CSC niche post-germination [52]. In the present study, seed exposure to sound waves caused an increase in the expression and activities *CYCB1;1* and *CYCD3;3* (Figure 3). Therefore, sound waves promote *Arabidopsis* root growth by increasing the expression of genes involved in cell division and consequently increasing cellular activity in the root meristem.

Root growth and development is also associated with hormone-mediated changes during cell division in the root meristem [53]. There was a significant decrease in the expression of cytokinin and ethylene signal transduction pathway genes in sound wave-treated seedlings, whereas there was a significant increase in the expression of auxin transport-related genes, except *AtAUX1* and *AtPIN2* (Figure 4). These changes would be expected to decrease the cytokinin content, while increasing the auxin content and inducing cell division in the root meristem, which was consistent with the results of the study. Blilou et al. [13] reported a decrease in the root length and meristem size of *pin1* and *pin2* single mutant *Arabidopsis* plants, and cell division inhibition in the root meristem of *pin3*, *pin4,* and *pin7* single mutants. In the present study, the root growth of *pin3,4,7* triple mutant and NPA-treated (auxin transport inhibitor) *Arabidopsis* was not significantly affected by sound waves (Figures S3 and S4), suggesting that the growth-promoting effect of sound was mediated by these PINs. We concluded that the expression of *PIN* genes was increased by the sound wave treatments, which activated auxin activities and consequently promoted *Arabidopsis* root growth.

Cell division, differentiation, and elongation of the root meristem are controlled by several hormones, such as auxin and cytokinin [7,8]. Ethylene also regulates primary root growth through crosstalk with auxin and cytokinin [20]. The production of IAA, as well as several other growth and defense hormones (GA, ABA, JA, and SA), is responsive to sound waves [33,34]. Moreover, the findings of our previous studies showed that sound waves can cause specific changes in the expression of ethylene biosynthesis-related genes [26,27,28]. In the present study, sound waves significantly affected the expression of cytokinin and auxin biosynthetic genes (Figure 5), causing a decrease in the cytokinin zeatin content of the roots and an increase in the auxin IAA content of the roots (Figure 6). Additionally, auxin biosynthesis inhibitor (yucasin) did not significantly affect the root growth of the sound wave-treated *Arabidopsis* plants (Figure S5). The expression of DR5*rev*:GFP was significantly higher in the roots of the *Arabidopsis* exposed to sound waves than that in the roots of the *Arabidopsis* in the control group (Figure 7). These results show that specific sound waves can increase the root auxin concentration by increasing the activity of auxin transporters as shown by the increased expression of *PIN1*, *3*, *4* and *7*. Also the expression of PIN2, an auxin transporter known to transport auxin toward the shoots was similar to untreated control suggests that auxin transport to shoots remain unchanged by the sound wave treatments. From this, it can be presumed that the auxin produced by specific sound wave treatment moves actively to the root tip, resulting in an increase in the auxin content, which increases the expression of the DR5 reporter at the root tip. These results suggest that sound waves can influence auxin biosynthesis, thereby regulating auxin content and promoting *Arabidopsis* root growth.

Auxin and cytokinin control root growth through antagonistic action. Auxin is distributed around the QC, which is maintained by a polar auxin transport system consisting of PINs and AUX1 [13]. PINs also mediate the distribution of auxins from shoots to roots, regulating cell division and cell expansion in the root meristem [14]. Cytokinin-related genes inhibit root growth by interfering with auxin transport genes in the root meristem and preventing the transfer of auxin [18]. Consistent with this, there was a significant increase in the root growth of the *Arabidopsis* cytokinin receptor mutants *ahk3-1* and *ahk4* (Figure S6). Contrarily, there was a reduction in the root growth of the auxin receptor single mutant *tir1-1*, with the *tir1-1 afb2-3* double mutant having similar root growth to that of the *Arabidopsis* in the control group (Figure S7).

Several factors function as cross-talk nodes between ethylene and auxin in *A. thaliana*. EIN3 and ERF1 bind directly to auxin-related gene promoters to regulate auxin accumulation in the root transition zone [22]. Particularly, PIF4 affects auxin-mediated growth by directly regulating the expression of auxin biosynthesis-related genes [38]. Significant reductions in the expression of ethylene biosynthesis-related genes have been observed in tomato plants exposed to sound waves [26]. Therefore, as Stepanova et al. [54] reported, it is presumed that a decrease in the expression of ethylene-related genes in *Arabidopsis* roots exposed to sound waves (Figure 4F) affects root growth by regulating the expression of genes related to auxin biosynthesis (Figure 5B). Other plant hormones are also involved in regulating root growth and have been reported to interact with each other to regulate root growth [19,20]. Future studies should examine the relationship between specific sound wave treatments and other hormones that affect plant growth. In addition, in order to investigate the effect of sound wave treatment at each growth stage, We will compare the effects of growth promotion by sound wave treatment in seedling and mature stage with seed-only treatment.

## 4. Materials and Methods

### 4.1. Plant Materials and Experimental Conditions

To investigate the effects of sound waves on *Arabidopsis thaliana* plants, wild type (Col-0) and mutant dry seeds in the Petri dish were exposed to 100 (single frequency) or 100 + 9k Hz (dual frequency) sound waves for 15-h periods on each of three consecutive days. The seeds were then sown on 1/2 MS-Gelrite plates containing 1% (*w*/*v*) sucrose and 0.5% (*w*/*v*) gelrite. Seedlings were grown vertically in a growth room (16 h light/8 h dark) at 22 ± 1 °C without sound waves to analyze root phenotypes. For sound waves, a single-frequency signal was generated using Pro Tools M-Powered software (Avid Technology, Burlington, MA, USA). The speaker volume was set at 80 dB. To block external noise, the sound wave treatments were performed in a custom-made, sound-proof chamber (Korea Scientific Technique Industry Co., Suwon, Korea). *Arabidopsis* seeds were placed in the sound-proof chambers to prevent the transfer of vibrations between samples during the sound wave treatments. The speaker model was a GENELEC 8010A (GENELEC, Lisalmi, Finland) with a frequency response between 74 and 20k Hz. For the untreated groups, the seeds were exposed to the same experimental conditions but without sound wave treatment. Primary root length was measured using an optical microscope (AXIOPLAN II; Carl Zeiss AG, Oberkochen, Germany). Sound-treated and untreated (control) root samples were immediately frozen in liquid nitrogen and stored at −80 °C. These samples were used for RNA extraction and hormone content analyses. All plant phenotypic images show representative individuals of at least 50 seedlings in three or more replicate experiments.

### 4.2. Propidium Iodide (PI) Staining

To monitor root growth, untreated and sound wave-treated seeds were sown in 1/2 MS medium and grown for 5 days. The roots were stained with PI (8 µg mL^–1^) for 5 min, and washed three times with distilled water, and then the cell number and length in the root apical meristem were determined using a confocal microscope (Leica DMi8 Microscope; Leica Microsystems, Wetzlar, Germany). The root apical meristem was determined from the QC to the first elongated cortex cells by observing cells using a confocal microscope [55]. The cell length was measured to determine the first elongated cortex cells. Root cell size was measured using ImageJ software (https://imagej.nih.gov/ij, accessed on 8 March 2021). This experiment was conducted in triplicate, and each experiment consisted of 25–30 seedlings.

### 4.3. Promoter-GUS Vector Construction and Transformation

Promoter-GUS vectors were constructed to confirm the expression of genes associated with cell elongation and division following sound wave treatment. The CaMV 35S promoter region of the pBI121 binary vector (Clontech, Mountain View, CA, USA) was removed using the restriction enzymes *Hin*dIII and *BamHI*, and the ~1-kbp promoter region of the two genes (*AtCYCB1;1* and *AtCYCD3;3*) of interest was inserted. The plasmid DNA of the cloned vector constructs was transformed into an *Agrobacterium* strain (GV3101) using 1 µg of plasmid DNA. *Agrobacterium* cells were grown to an OD_600_ of 0.8–1.0 and transformed into 5-week-old *Arabidopsis thaliana* plants using floral dip method [56]. For each construct, 25–30 independent transgenic lines were obtained in the T1 generation by kanamycin (50 mg/mL) selection. To confirm single-copy transgene insertion, approximately 120 seeds of each T2 transgenic line were sprinkled onto MS plates containing kanamycin, and lines with a segregation ratio of 3:1 were selected. GUS activity in each transgenic plant was analyzed using three to five independent lines of homozygous T3 plants.

### 4.4. Histochemical GUS Assay Following Sound Wave Treatment

The *Arabidopsis* transgenic seeds expressing *GUS* under the control of the promoters described in the text were treated with sound waves over a three-day period as described above and then sown in 1/2 MS medium and grown vertically for 5 days. Transgenic seedlings were subjected to GUS staining using X-Gluc solution (3 mM 5-bromo-4-chloro-3-indolyl-β-glucuronide in 100 mM sodium phosphate, 0.5 μM K3[Fe(CN)6], 0.5 μM K4[Fe(CN)6], 10 mM EDTA, and 1% TritonX-100) (Sigma-Aldrich, St. Louis, MO, USA) and stored at 37 °C in the dark for one day. The next day, the chlorophyll was slowly removed from the samples by replacing the solution with a series of ethanol solutions (50%, 75%, and 100%). GUS activity was then assessed using an optical microscope (Leica DM5500 B; Leica Microsystems). This experiment was conducted in triplicate, and each experiment consisted of 25–30 seedlings.

### 4.5. RNA Extraction and Quantitative Real-Time PCR (qPCR)

Three independent biological replicates were used for each experiment. Roots from the 5-day-old seedlings were harvested and immediately frozen in liquid nitrogen. Frozen tissue was ground to a powder in liquid nitrogen using a mortar and pestle. Total RNA was extracted using a Plant RNeasy Extraction Kit (Qiagen, Hilden, Germany). RNA samples were treated with DNase I (Qiagen), and cDNA was synthesized using an amfiRivert Platinum cDNA Synthesis Master Mix (GenDEPOT, Barker, TX, USA). Quantitative real-time PCR (qPCR) analysis was performed using an *AccuPower* *2X* GreenStar qPCR Master Mix (Bioneer, Daejeon, Korea) and the CFX96 Touch Real-Time PCR Detection System (Bio-Rad, Hercules, CA, USA). The relative mRNA levels were determined by normalizing the PCR threshold cycle number of each target gene with that of the *Actin2* reference gene. Three technical replicates were performed for each biological replicate analyzed. The primers used for qPCR analysis are shown in Table S1.

### 4.6. LC-MS and Conditions for Hormone Content Quantification

An Agilent 6410 B6410B Triple Quadrupole LC/MS (Agilent Technologies, Santa Clara, CA, USA) equipped with an electrospray ionization (ESI) source was employed for the analysis. Indole acetic acid (IAA) as auxin and *trans-zeatin* (zeatin free base form) as cytokinin were purchased from Sigma-Aldrich and used as a reference standard. Then, 0.1 g of each sample was mixed with 1 mL of 75% ethanol and centrifuged at 2500 rpm for 10 min. Aliquots of 5 µL of the processed samples were injected into the HPLC system (1200 Series LC; Agilent Technologies) fitted with a Kinetex C8 2.6 µm 80 Å 50 × 2.1 mm column (Phenomenex, Torrance, CA, USA) maintained at 35 °C. The ESI was operated at +3000 V and a source temperature of 380 °C. The capillary voltage, cone voltage, and source offset were set to 3 kV, 30 kV, and 30 V, respectively. The flow rates for the desolvation and cone gases were set to 650 and 150 L/h, respectively, with a nebulizer pressure of 15 bar. A mobile phase composed of 0.1% formic acid in distilled water (Buffer A) and 0.1% formic acid in acetonitrile (Buffer B) was used to separate the analytes and pumped into the ESI chamber at a flow rate of 0.5 mL/min for 20 min. The fragmentor voltage and collision voltage were set at 70 V. Ions were detected in the multiple-reaction monitoring mode by monitoring the transition pairs of m/z 176 → 130 (IAA) and 220 → 136 (Zeatin). Data were acquired using the MassHunter software (Version B.04.00).

### 4.7. GFP Fluorescence Analysis

A *DR5rev:GFP* reporter was used to analyze auxin responses in the roots [13,57]. The seeds were treated with sound waves, and then the seeds were grown vertically on half-strength MS medium containing 1% (*w*/*v*) sucrose and 0.5% (*w*/*v*) gelrite in a growth room (16 h light/8 h dark) at 22 ± 1 °C. On the 5th day after sowing, GFP fluorescence intensities at the root tip were measured using a confocal microscope. For quantification of fluorescence intensities, we used ImageJ software (NIH, Bethesda, MD, USA). This experiment was repeated three times, and each replicate had at least 20–25 seedlings.

### 4.8. Auxin Transport and Biosynthesis Inhibitor Treatment

Sound-wave-treated seeds were sown on half-strength MS medium containing 20 or 50 μM N-1-naphthylphthalamic acid (NPA; Auxin transport inhibitor; Sigma-Aldrich) and 5-(4-chlorophenyl)-4H-1,2,4-triazole-3-thiol (yucasin; auxin biosynthesis inhibitor; Carbosynth, Berkshire, UK), respectively, and then grown vertically in a growth room (16 h light/8 h dark) at 22 ± 1 °C. On the 5th day after sowing, the primary root length was measured using an optical microscope. This experiment was repeated three times, and each replicate had at least 25–30 seedlings.

### 4.9. Statistical Analyses

Analysis of variance was performed using the Statistical Package for Social Science (SPSS, version 25.0; IBM Corporation, Armonk, NY, USA), and a Duncan’s multiple range test was used to determine the statistical significance of the means at *p* < 0.05.

## 5. Conclusions

In conclusion, the findings of this study showed that exposure to specific sound waves can promote root growth in *Arabidopsis* seedlings by inducing the development of the root meristem through the activities of phytohormones, particularly auxin and cytokinin. This sound waves treatment technology is thought to be able to replace the use of artificial synthetic fertilizers and agricultural chemicals that are currently being used to promote the growth of crops. Consequently, this is expected to be very useful technology for maintaining an eco-friendly and sustainable agricultural ecosystem in the future.

## Data Availability

Requests for further information about resources, reagents and data availability should be directed to the corresponding author.

## Supplementary Materials

The following are available online at https://www.mdpi.com/article/10.3390/ijms22115739/s1, Figure S1: Promotion of root growth in pumpkin by 9 kHz sound wave treatment. Images show representative individuals of at least 30 seedlings in three or more replicate experiments., Figure S2: Selection of frequency to promote root growth in *Arabidopsis thaliana*. Seeds were treated with 100, 500, and 9k Hz sound waves for 15 h, and 100 + 9k Hz and 500 + 9k Hz sound waves for 5 h or 15 h for (A) 3 d and (B) 5 d, and seedlings were observed on the 5th day after sowing using an optical microscope (×10). Scale bars represent 2 mm. Images show representative individuals of at least 50 seedlings in three or more replicate experiments. Different letters above the bars indicate significantly different mean values (*p* < 0.05) based on Duncan’s multiple range test., Figure S3: Effect of sound wave treatments on the root growth of *Arabidopsis* wild-type (Col-0) under auxin transport inhibitor naphthylphthalamic acid (NPA) conditions. (A) 20 µM and (B) 50 µM NPA. An optical microscope was used to observe root growth (×10) and root tips (× 60). Scale bars represent 1 mm (Top) and 200 µm (Bottom). (C) Primary root and root tip length measurement for each treatment (±standard error). Different letters indicate significantly different mean values (*p* < 0.05) based on Duncan’s multiple range test. This experiment was conducted in triplicate, and each experiment measured 25–30 seedlings., Figure S4: Effect of sound wave treatments on root growth of wild-type (Col-0) and *pin3 pin4 pin7* triple mutant *Arabidopsis thaliana*. (A) An optical microscope was used to observe root growth (×10) and root tips (×60). Scale bars represent 2 mm (A) and 200 µm (B). (C) Primary root and root tip length measurement for each treatment (±standard error). Different letters indicate significantly different mean values (*p* < 0.05) based on Duncan’s multiple range test. This experiment was conducted in triplicate, and each experiment measured 25–30 seedlings, Figure S5: Effect of sound wave treatments on the root growth of *Arabidopsis* wild-type (Col-0) under auxin biosynthesis inhibitor (yucasin) conditions. (A) 20 µM and (B) 50 µM yucasin. An optical microscope was used to observe root growth (×10) and root tips (×60). Scale bars represent 1 mm (Top) and 200 µm (Bottom). (C) Primary root and root tip length measurement for each treatment (±standard error). Different letters indicate significantly different mean values (*p* < 0.05) based on Duncan’s multiple range test. This experiment was conducted in triplicate, and each experiment measured 25–30 seedlings., Figure S6: Effect of sound wave treatments on root growth of wild-type (Col-0), and ahk3-1 and ahk4 mutant *Arabidopsis thaliana*. (A) An optical microscope was used to observe root growth (×10) and root tips (×60). Scale bars represent 2 mm (A) and 200 µm (B). (C) Primary root and root tip length measurement for each treatment (±standard error). Different letters indicate significantly different mean values (*p* < 0.05) based on Duncan’s multiple range test. This experiment was conducted in triplicate, and each experiment measured 25–30 seedlings., Figure S7: Effect of sound wave treatments on root growth of wild-type (Col-0), *tir1-1* single mutant, and *tir1-1 afb2-3* double mutant *Arabidopsis thaliana*. (A) An optical microscope was used to observe the growth of roots (× 10) and root tips (×60). Scale bars represent 2 mm (A) and 200 µm (B). (C) Primary root and root tip length measurement for each treatment (±standard error). Different letters indicate significantly different mean values (*p* < 0.05) based on Duncan’s multiple range test. This experiment was conducted in triplicate, and each experiment measured 25–30 seedlings, Table S1: qRT-PCR primer sequences used in this study.

## Abbreviations

Hz	Hertz
dB	Decibel
PI	Propidium iodide
GUS	β-glucuronidase
QC	Quiescent center
TZ	Transition zone
EZ	Elongation zone
qRT-PCR	Quantitative real-time polymerase chain reaction
NPA	N-1-naphthylphthalamic acid
Yucasin	5-(4-chlorophenyl)-4H-1,2,4-triazole-3-thiol
CK	Cytokinin
IAA	Indole acetic acid
ABA	Abscisic acid
GA	Gibberellin
JA	Jasmonic acid
SA	Salicylic acid
GFP	Green fluorescent protein
