# Supplementary material for: Sound Waves Promote Arabidopsis thaliana Root Growth by Regulating Root Phytohormone Content
Source: Int J Mol Sci. 2021 May 27;22(11):5739. doi: 10.3390/ijms22115739 (PMC8199107; doi:10.3390/ijms22115739)
Supplement: Supplementary file 1 [file ijms-22-05739-s001.zip › Supplementary Table S1.pdf]

Table S1. qPCR primer sequences used in this study.

Gene	Sequence
**AtACT2-F**	GGTAACATTGTGCTCAGTGGTGG
**AtACT2-R**	AACGACCTTAATCTTCATGCTGC
**AtAHK2-F**	TTATGCAAGCCGACAGTTCCACA
**AtAHK2-R**	CTATCCCGGGCTCACTCACAAAC
**AtAHK3-F**	AGAGGGAAGAGTTCGAGAGGCAA
**AtAHK3-R**	CTGAGCAAAGATGACTGGAGCGT
**AtAHK4-F**	CTCGGAATGCTTGCTATGCTCCT
**AtAHK4-R**	CTTTCCAGCTTCAATCTTGGCGC
**AtARR1-F**	AACATAGAGGAGGTGGTGGTGGT
**AtARR1-R**	TCCTTGCTCTTCTCCTTCCTCGT
**AtARR10-F**	GGCGTTGAGAGAGCTGTTCCAAA
**AtARR10-R**	GCCCTGTTAGCTTGTTGAATGGC
**AtARR12-F**	CAGCCTTTCTTCTCCTCCTGCAG
**AtARR12-R**	AAGCTGGTCGAACTCTAAAGGCA
**AtSHY2-F**	GGCTCAGATTGTTGGATGGCC
**AtSHY2-R**	TTTCCTCAAGTATGGTGCACCATC
**AtPIN1-F**	TACTCCGAGACCTTCCAACTACG
**AtPIN1-R**	TCCACCGCCACCACTTCC
**AtPIN2-F**	GGCGAAGAAAGCAGGAAGA
**AtPIN2-R**	GGTGGGTACGACGGAACA
**AtPIN3-F**	CGGAGCACCTGACAACGAT
**AtPIN3-R**	CGGATCTCTTTAGCACCTTGGT
**AtPIN4-F**	CAACCCAAAATCATTGCTTGTG
**AtPIN4-R**	CGGACCGGTTATAAATCTGACC
**AtPIN7-F**	ACTCCTCGTCCGTCTAATCTCAC
**AtPIN7-R**	GAAGCCATAGCACAACTCTCCTC
**AtAUX1-F**	CCGAGCTGGACGGCGATGTACG
**AtAUX1-R**	CATGTGCGGCGGCTGCAGCT
**AtLAX2-F**	GGACTGGGGCATTCACGATCA
**AtLAX2-R**	GGTGGTGGGCATTGGTAGCAT
**AtLAX3-F**	CATATTTGGGGCGTGCTGTGC
**AtLAX3-R**	CACATCCTCAGCCTGGCCATG
**AtEIL1-F**	GGACACGACTCTTGGTTCGCT
**AtEIL1-R**	GGCCACCACTCTTCATTCCCA
**AtEIN2-F**	AGTGACTGGGCTGGTGGTTTG
**AtEIN2-R**	GCTTCCGCTCTGTTACTCGCA
**AtEIN3-F**	ACATGGTGGAAGGAAGTT
**AtEIN3-R**	TTGCCGCTACTGTTATTG
**AtERF1-F**	ATTCTTTCTCATCCTCTTCTTCT
**AtERF1-R**	CGAATCTCTTATCTCCGCCG
**AtPIF4-F**	CCAGATCATCTCCGACCGGTTTG
**AtPIF4-R**	CTAGTGGTCCAAACGAGAACCGT

**AtCYCB1;1-F**	ATGTCCGATTCGAGCTAAACCCG
**AtCYCB1;1-R**	GCCGACATGAGAAGAGCACTGAG
**AtCYCD1;1-F**	AGCGAGCTTTCTTGAGTACTGGC
**AtCYCD1;1-R**	GCTCAATCCGTCACACCAAGTCT
**AtCYCD3;3-F**	CTGCTAAGGTTGAAGAGATCCGTGT
**AtCYCD3;3-R**	GATGCATCCTCCAGTCAAGAGTAGA
**AtIPT1-F**	AGAGATCACAACGAATCAGATTACGT
**AtIPT1-R**	ATGACGCCGAGGAGATGGT
**AtIPT3-F**	CATGGCGAATCTCTCCATTGA
**AtIPT3-R**	AGTTGGAACCTCCAACGATGA
**AtIPT5-F**	AGGATTTTCAGCGTGAAGCAA
**AtIPT5-R**	CTATGATCGGGACACGGTCTCT
**AtIPT7-F**	AACCTAACGGCCACCCAGTA
**AtIPT7-R**	TGTTGTTCGCTGAGAGTTTCGA
**AtCYP735A1-F**	GGCCATGGTTTCGCAATC
**AtCYP735A1-R**	CCGTTTCCGTTAAGCAAAGC
**AtCYP735A2-F**	ATGGTGTCCCTTCCGTTGAACA
**AtCYP735A2-R**	GAGGGTAAAGTCTTAATGACTCGT
**AtTAA1-F**	TGGTGATCCAACGGCGTACG
**AtTAA1-R**	CGTGGCCTGTGATCTTGGAG
**AtYUC1-F**	CAAAAACGAGTTCTTGGCCTA
**AtYUC1-R**	CCAAACGAAAGATTGGCCATCAATAAC
**AtYUC6-F**	GGTAAACTAGCACATGACCACC
**AtYUC6-R**	CAAACTTATCCAACCCCTCAAACC
