# Supplementary figures and images for: Sound Waves Promote Arabidopsis thaliana Root Growth by Regulating Root Phytohormone Content
Source: Int J Mol Sci. 2021 May 27;22(11):5739. doi: 10.3390/ijms22115739 (PMC8199107; doi:10.3390/ijms22115739)

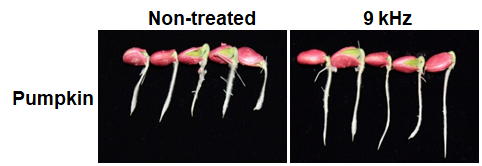

Supplement: Supplementary file 1 [file ijms-22-05739-s001.zip › S1.tif]

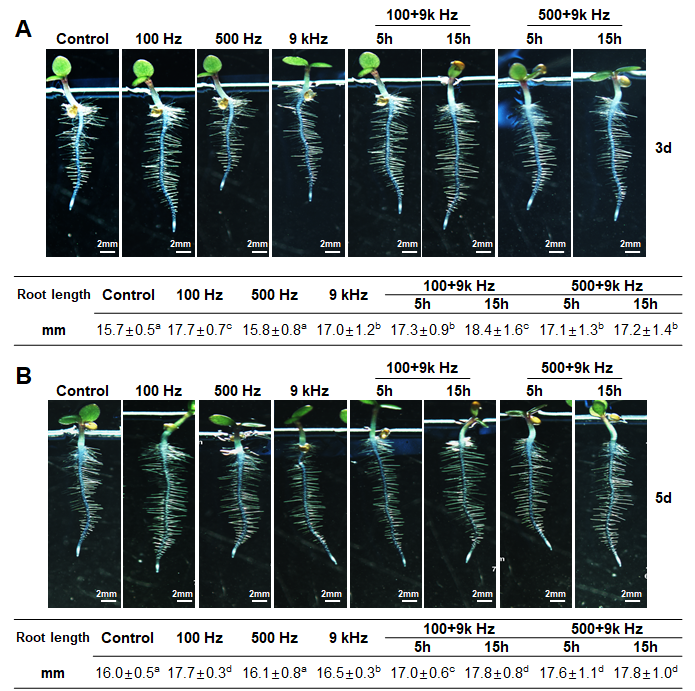

Supplement: Supplementary file 1 [file ijms-22-05739-s001.zip › S2.tif]

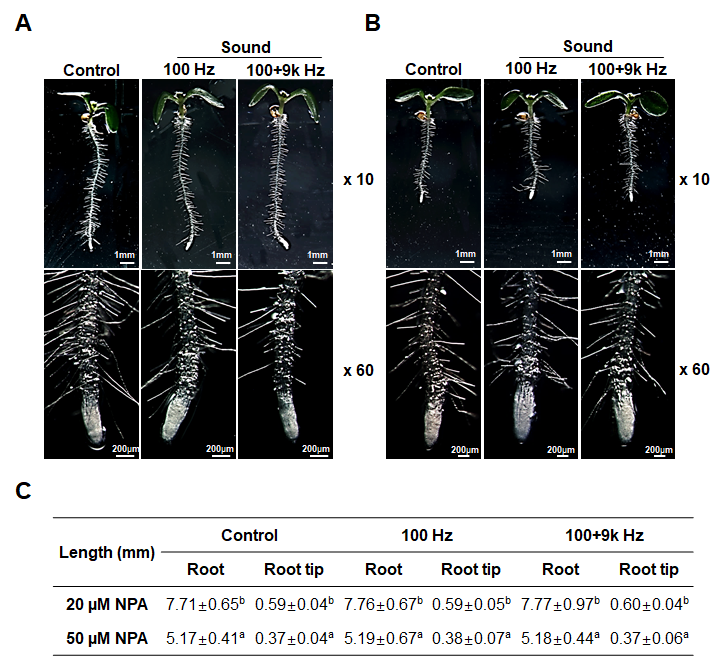

Supplement: Supplementary file 1 [file ijms-22-05739-s001.zip › S3.tif]

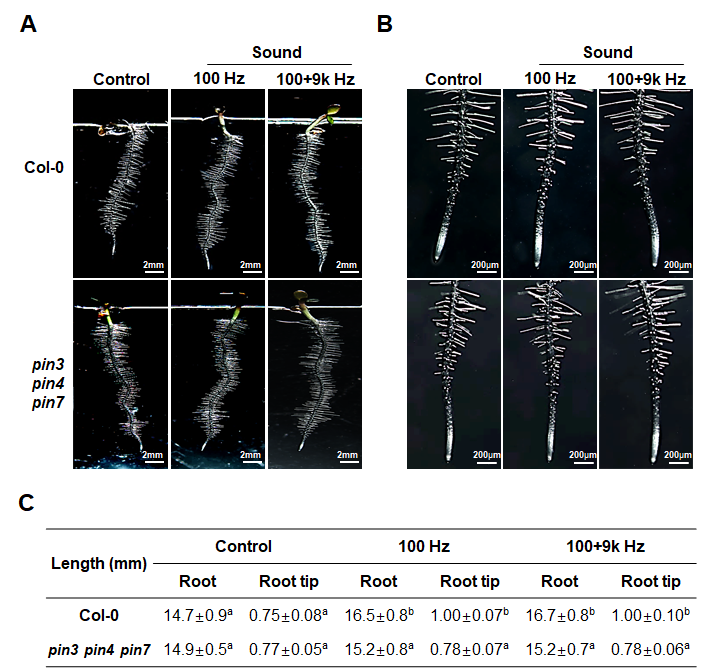

Supplement: Supplementary file 1 [file ijms-22-05739-s001.zip › S4.tif]

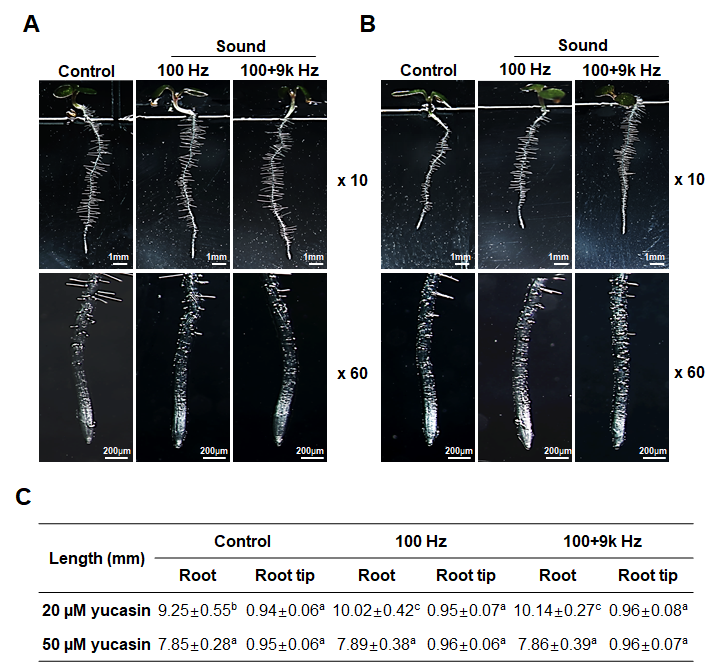

Supplement: Supplementary file 1 [file ijms-22-05739-s001.zip › S5.tif]

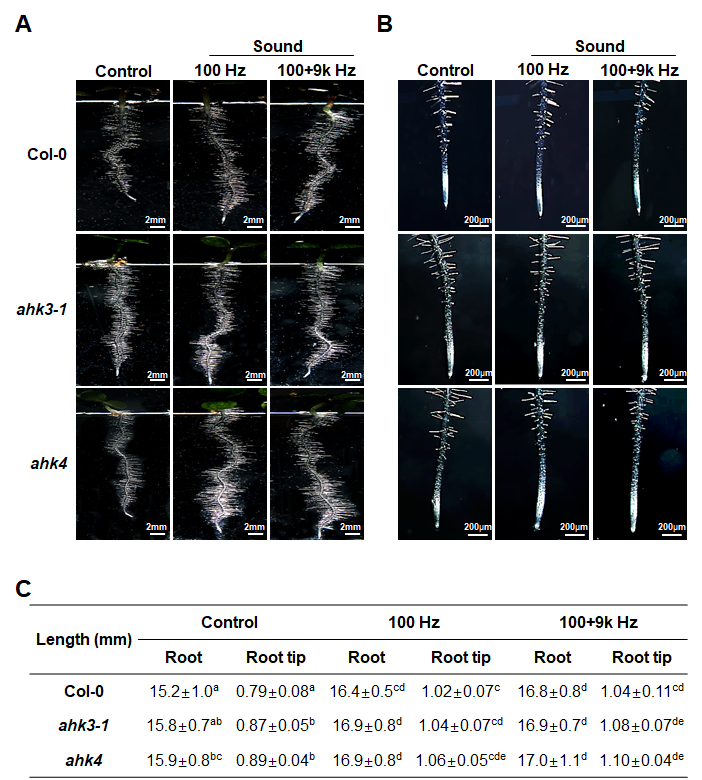

Supplement: Supplementary file 1 [file ijms-22-05739-s001.zip › S6.tif]

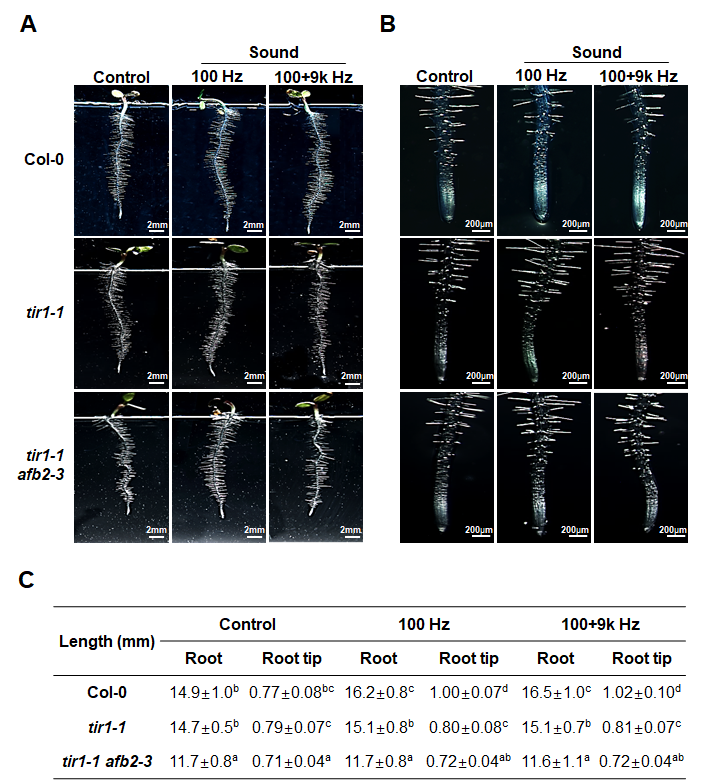

Supplement: Supplementary file 1 [file ijms-22-05739-s001.zip › S7.tif]
